# Aging Adults’ Motivation to Use Embodied Conversational Agents in Instrumental Activities of Daily Living: Results of Latent Profile Analysis

**DOI:** 10.3390/ijerph19042373

**Published:** 2022-02-18

**Authors:** Urška Smrke, Nejc Plohl, Izidor Mlakar

**Affiliations:** 1 Faculty of Electrical Engineering and Computer Science, University of Maribor, Koroška Cesta 46, 2000 Maribor, Slovenia; urska.smrke@um.si; 2 Department of Psychology, Faculty of Arts, University of Maribor, Koroška Cesta 160, 2000 Maribor, Slovenia; nejc.plohl1@um.si

**Keywords:** instrumental activities of daily living, embodied conversational agents, latent profile analysis, ageing adults, personalized assistive technology

## Abstract

The rapidly increasing share of ageing adults in the population drives the need and interest in assistive technology, as it has the potential to support ageing individuals in living independently and safely. However, technological development rarely reflects how needs, preferences, and interests develop in different ways while ageing. It often follows the strategy of “what is possible” rather than “what is needed” and “what preferred”. As part of personalized assistive technology, embodied conversational agents (ECAs) can offer mechanisms to adapt the technological advances with the stakeholders’ expectations. The present study explored the motivation among ageing adults regarding technology use in multiple domains of activities of daily living. Participants responded to the questionnaire on the perceived importance of instrumental activities of daily living and acceptance of the idea of using ECAs to support them. Latent profile analysis revealed four profiles regarding the motivation to use ECAs (i.e., a low motivation profile, two selective motivation profiles with an emphasis on physical and psychological well-being, and a high motivation profile). Profiles were compared in terms of their acceptance of ECA usage in various life domains. The results increase the knowledge needed in the development of assistive technology adapted to the expectations of ageing adults.

## 1. Introduction

We are facing a rapid increase in the share of ageing adults in the global population. Specifically, people aged 65 and above are estimated to represent 16% of the total world population in 2050, compared to 9% in 2019 [1]. This social change will likely have important implications for all sectors of society, especially health and other types of services for the ageing population [2,3]. As the need for these services becomes even more pronounced, new approaches are needed to help mitigate the pressures these sectors are facing. One of the recognized strategies is the development of effective assistive technology, as it has the potential to support the ageing individuals in living independently and safely in the environment of their choice, contributing to their general quality of life, as well as their physical and mental health [4,5,6].

General technological advances have led to the expansion of areas that can be addressed with digital services, accompanied by the increasing complexity of the underlying user interfaces and the use of such services [7]. However, the currently available technology developed to assist ageing individuals in their daily lives generally poorly reflects the variability of needs, preferences, and interests that develop during ageing [8] and results in a very heterogeneous population category [4]. It also fails to reflect how individuals’ resources and preferences—including ICT literacy and social and economic resources—decline as a natural consequence of ageing [8]. The current approach to the development of technology, hence, often focuses on what is possible rather than what is needed and preferred. Therefore, focusing the research on the latter is needed to develop embodied conversational agents (ECAs) better equipped to meet the expectations of their users and better support the ageing population in autonomous and independent daily living [9].

### 1.1. Ageing and Instrumental Activities of Daily Living

The process of ageing brings an increased risk for many challenges and impairments in various areas of functioning, such as physical (e.g., chronic conditions, functional limitations and disabilities reflected in difficulty in managing daily tasks and limited mobility), sensory and cognitive (e.g., memory-related changes), as well as behavioral (e.g., changed sleep patterns) and psychological (e.g., the general decline of quality of life, social isolation, loneliness) [5,10,11]. These have a considerable impact on the individual’s competence to perform activities essential for maintaining independent living and active ageing. With the increasing age of the population [4], the share of individuals with a heightened risk for vulnerability and loss of independence also increases [10,11].

The potential to live independently depends on the functional capacity of a person (also known as everyday competence), and, as such, has substantial effects on their quality of life [11,12]. When this capacity is diminished, it results in increased difficulty performing daily activities and the need for additional support. Poor performance in daily activities is linked to disability [11] and predicts mild cognitive impairment, dementia, and mortality [10,13].

When severe impairments in functional capacity occur, they negatively affect the performance of activities of daily living (ADLs), e.g., bathing, dressing, and feeding, which are fundamental for independent living. Such a severe impairment reflected in ADLs is most often preceded by the milder limitations in functional capacity, predominantly impairing the performance of instrumental activities of daily living (IADLs), e.g., shopping, preparing meals, handling finances, which are central to maintaining autonomy [13] and are the focus of the present study. Compared to ADLs that refer to basic and essential human functions, IADLs generally refer to more complex adaptive behaviors [14]. As such, IADLs are easier to address with (conversational) technology; for example, people who cannot feed themselves need human interaction and help to a larger degree than people who simply need some support preparing their meals. As difficulties performing IADLs typically occur before difficulties with ADLs, they are also more prevalent. A large-sample survey of ageing adults in Spain reports that disability for IADLs—defined as having a lot of difficulty or being unable to perform at least one activity measured by the Lawton’s questionnaire (e.g., preparing own meals)—is present in 31.9% of the sample, compared to 11.1% in the case of disability for ADLs [11]. Similarly, at a European level, 23.8% of adults aged 65 and more exhibit a limitation in at least one IADL [15]. Such disability and limitations in IADLs may signalize higher-order cognitive impairments. However, it is important to note that the performance of such activities does not necessarily exhibit linear decline over time but rather shows a dynamic pattern of decline and recovery [13], which implies that the performance in IADLs can be sustained or even improved [11].

### 1.2. Embodied Conversational Agents

ECAs are virtual entities that can convey messages and engage in conversation in a human-like manner by using speech, language, gestures, and facial expressions [16]. While verbal channels carry symbolic/semantic interpretation of the message, the non-verbal channels (i.e., gestures, facial expressions, and prosody) serve as an orchestrator of communication and are integral to comprehension [17,18]. By incorporating features such as facial expressions and gestures, social links between humans and their virtual counterparts may be established. Such links may simplify interaction and induce emotions [19]. Overall, ECAs exhibit the potential to aid in patient care [20] and may improve the performance of ageing individuals in IADLs by taking the role of a social companion, coach, or medical assistant that provides company and information [21]. In clinical settings, they have already been used to support activities in physical health (e.g., adherence to treatment, diet, and exercise behaviors) as well as psychological and social health (e.g., depression [22]; dementia [23]). Although scarce and in its early stages, the existing research generally also shows promising results related to their effectiveness for ageing adults (e.g., [24,25]). Despite the potential benefits, the currently available technologies are not widely adopted, and interest in them usually fades after the initial stages, especially among older adults [26]. This problem could be at least partially attributed to the relatively poor understanding of older adults’ digital practices, design preferences, and attitudes toward the adoption of ECAs [27]. 

While individual-level barriers to the adoption of new technologies, such as technology-related stress/anxiety, lack of trust, and low technological self-efficacy [28,29,30,31], are disproportionally pronounced in older adults compared to the other age groups [26], we argue that a different approach to the development of technology could help mitigate these barriers. Specifically, the current approach to the development of aiding technology exhibits several opportunities for improvement. First, assistive technology for aging adults is often developed from the perspective on ageing that is paternalistic, medical, and instrumental, focusing primarily on negative aspects of ageing [2]. While such a perspective has benefits, as it facilitates the development of technology that can mitigate the critical aging-related difficulties, it is somewhat narrow, as it overlooks the potential of technology to support aging adults in other domains and increase their quality of life. Second, the proposed solutions are most often tailored based on the existing and available technologies rather than based on various stakeholders’ needs [4]. Hence, as Sayago [2] proposes, the current approach is limited by its inability to perceive the complexity and diversity of the process of ageing and its inability to fully understand the various possibilities by which digital assistive technology could support and empower ageing adults.

### 1.3. The Need for a Personalized Approach

Even though the scientific literature recognizes the critical role of end-users’ needs, there is a lack of systematic investigation on how the proposed technological solutions could take them into account [4]. As in the process of ageing, "rather than becoming a homogenous category to be designed for, we grow more diverse rather than less, by virtue of our different life experiences in different bodies" [32] (p. 3921), the approach which grants more room to the analysis of differing characteristics of the ageing population is needed. Such an approach would more adequately address the diversity of needs, preferences, and interests of ageing individuals, leading to technology that is more adequately suited to support and empower them and is hence more effective [9]. An example of such an approach is a study by Balog and colleagues [33], which highlights the importance of examining the diversity of needs and preferences of elderly patients. In particular, the researchers found two distinct profiles related to the use of ambient assisted living technology. However, the authors note that their study had a few methodological issues, such as a small sample size (the sample consisted of 62 aging adults; [33]). Another example is presented in a study by Santini and colleagues [34], which emphasizes the value of end-users’ inputs and personalization in the development of an ECA-based virtual coach for active and healthy ageing. 

The current body of knowledge lacks user-centered research focusing on conversational agents, especially when it comes to understanding the motivation for using such technology [23]. As the diverse process of ageing is reflected in diverse patterns of technology adoption [35], exploring the motivation could provide vital information for the development of conversational agents that are tailored to the needs of ageing adults and, as such, can support their performance in IADLs more adequately. For example, while some individuals may need and desire help handling finances and would thus likely be motivated to use ECAs that address this aspect, others may find ECAs focusing on such services irrelevant. To avoid this misfit between the needs and preferences on the one hand and the developed technology on the other, we first need to understand the heterogeneity of needs and preferences and then develop more personalized solutions.

### 1.4. The Present Research

The present preliminary research aims to examine the profiles of ageing adults regarding the importance of IADLs in their daily lives as a way of exploring their motivation to use ECAs in different life domains. We employ a person-centered approach to analysis by using a latent profile analysis (LPA) procedure to unravel the groups of individuals that differ in their perception of specific areas of IADLs. We will further validate these profiles by comparing the participants’ acceptance of ECAs in the corresponding areas. The findings of this study are expected to help increase the critical knowledge, important in developing the assistive technology adapted to the specific needs, preferences, and interests of ageing individuals. Specifically, identified profiles of ageing adults regarding the perception of IADL areas and motivation to use ECAs may represent a foundation for further studies on the development of assistive technologies.

## 2. Materials and Methods 

### 2.1. Participants

Data collection took place between November 2018 and November 2019. In the first stage, we contacted the major Slovenian residential care facilities (also known as retirement homes). The institutions that decided to participate directed us towards aging adults eligible for taking part in our study (i.e., individuals without dementia or other notable incapacities). In the second stage, such participants were individually invited to participate in the study. Those who expressed their willingness to participate were asked to sign informed consent. 

A total of 189 older adults participated in the study, but the data of four participants (2.1%) had to be excluded from the analyses due to missing crucial demographic data. The final sample thus consists of 185 older adults aged 65–97 years (*M* = 81.42, *SD* = 7.84), the majority of whom were female (*n* = 122; 65.9%). At the time of the study, most of the sample lived in residential care settings (*n* = 182; 98.4%).

### 2.2. Measures

The questionnaire employed in this study was adapted from the widely used Lawton instrumental activities of daily living scale [36,37]. Lawton’s questionnaire was developed for the functional assessment of the elderly, i.e., for the assessment of their ability to perform instrumental activities of daily living, and is mainly used in the assessment of the rehabilitation needs of an individual, in the planning of the specific services a person might need, etc. It assesses eight areas: the ability to use a telephone, shopping, food preparation, housekeeping, laundry, (mode of) transportation, responsibility for own medication or medicine administration, and ability to handle finance [36]. In the present study, areas assessed were slightly updated regarding the envisioned possibilities of employing the assistance of ECAs, resulting in the following list of activities: general technology, communication, food, health, shopping, money, and infotainment. Since the aim of this study does not require the assessment of the functional ability of an individual as such, but the importance of individual areas for them and their view on the acceptability of using ECAs in these areas, the questionnaire was constructed in a way that participants rated the importance of each area (i.e., “*Rate the importance of the following activities in your daily life:*”) on a 10-point scale ranging from one (“*unimportant*”) to 10 (“*extremely important*”). IADL areas were presented with a short description in the questionnaire. The second set of questions required participants to indicate their acceptance of the idea of using an ECA in each of the assessed IADL areas (e.g., “*Would you be willing to use an embodied conversational agent to help you with your purchases*?”) on a five-point scale from one (“*unacceptable*”) to five (“*great”*).

The questionnaire was administered in a paper-pencil format in the presence of a researcher who was available for explanations of lesser-known concepts when needed.

### 2.3. Procedure and Statistical Analyses

The primary goal of the analyses was to identify motivationally distinct profiles of older adults and investigate how they differ in acceptance of ECAs in different areas of daily living. The profiles were derived through LPA, with participants’ answers to seven items capturing the importance of various activities (see Section 2.2), gender, and age used as indicators. Once the best fitting solution out of two through five profile models was found, we compared the identified profiles with separate analysis of variance (ANOVA) tests.

We conducted LPA analyses using MPlus 8.0 [38] and used maximum likelihood with robust standard errors (MLR) as the estimator. Our models were compared using various fit indices. First, relative fit information criteria, specifically the Bayesian Information Criterion (*BIC*) and the Sample-Adjusted BIC (*SABIC*), which helps to reduce the typical BIC sample size penalty [39], were used. In the case of *BIC* and *SABIC*, lower values indicate a better fit [40]. Second, we used entropy as an indicator of classification quality (i.e., confidence with which individuals have been correctly classified as belonging to one profile or another). Entropy values can range from 0 to 1, with higher values (generally those above 0.80) indicating satisfactory classification quality [41]. Third, statistical comparisons between a model with *k* profiles and a model with *k*−1 profiles were performed using the Bootstrapped Likelihood Ratio Test (*BLRT*). A significant *p*-value suggests that the model of interest provides a better fit compared to a model with one fewer profile [42]. Final model selection was additionally based on parsimony, meaningfulness (e.g., profile sizes), and theoretical plausibility [43,44,45].

The extracted profiles were then compared using the IBM SPSS Statistics 26.0 software. Specifically, we conducted several analysis of variance (ANOVA) tests to determine whether the motivationally distinct profiles differ in their acceptance of ECAs in different IADL domains. Welch’s ANOVA was used instead of ANOVA in cases where the data exhibited unequal variances. Significant results were further explored with post-hoc tests (Hochberg’s GT2 correction or Games–Howell correction, depending on the homogeneity of variance assumption).

## 3. Results

We present means, standard deviations, and correlations of all indicator variables in Table 1 below. In general, areas that participants found most important are health, communication, and infotainment, while food, shopping, and managing money were assessed as least important. As can be seen in the table, correlations between pairs of indicators were generally low; the strongest relationship, which can be categorized as intermediate, was observed between technology importance and communication importance (*r* = 0.45 **), followed by the correlation between technology importance and infotainment importance (*r* = 0.40 **).

### 3.1. Identification and Interpretation of Profiles

In the next step, we investigated the number and interpretation of profiles. As noted in the Methods section, several fit statistics were considered to evaluate different profile solutions. They are displayed in Table 2. Among the relative fit information criteria, *BIC* suggests the extraction of four profiles, while *SABIC* shows a better fit in the case of the five-profile solution. However, the five-profile solution also exhibits the lowest (yet satisfactory) entropy, a non-significant *BLRT* test, and a profile containing only about 5% of the sample. Hence, the results predominantly favor the extraction of four profiles.

**Figure 1:**
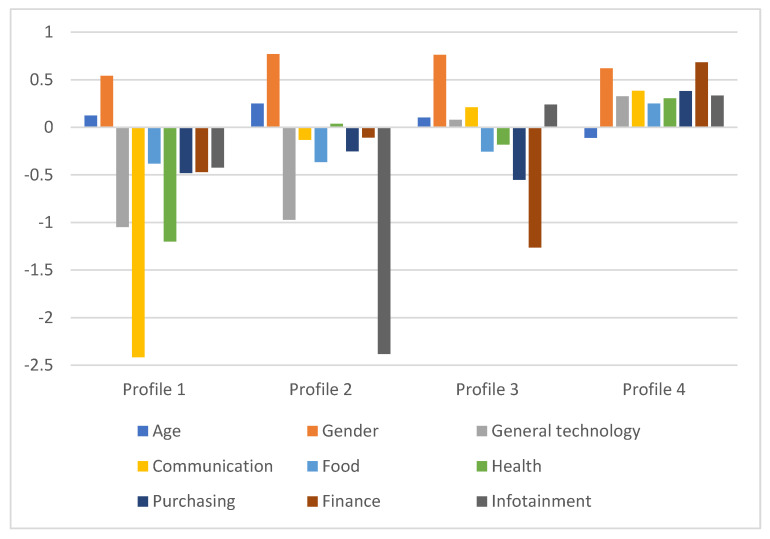
Characteristics of extracted profiles. The figure contains absolute values for gender and standardized (*Z*) values for other variables.

The first extracted profile contains 19 participants (10.3% of the sample), who are generally older and perceive all measured activities, especially communication, as unimportant. This group can be labelled as a low motivation profile. The second profile contains 17 participants (9.2% of the sample). The members of this group are predominantly female and comparatively older than other participants. They are also characterized by the low importance of most measured activities, especially infotainment, but exhibit an above-average perceived importance of health. As such, they can be labelled as a selective motivation profile that appreciates physical well-being. The third profile is larger than the previous ones and contains 47 participants (25.4%), most of whom are female and older than the average participant. These participants, again, exhibit low perceived importance of most measured activities. However, compared to other profiles, they show higher importance of technology, communication, and infotainment. Hence, they can be labelled as a selective motivation profile that appreciates psychological well-being. The last (and the largest) profile consists of 102 younger participants (55.1%). As opposed to the previous profiles, this profile exhibits a high importance of all measured activities. Differences between the profiles are illustrated in Figure 1.

### 3.2. Validation of the Extracted Profiles

To validate the selected LPA solution and the four extracted profiles, we compared these profiles regarding the variables related to the acceptability of the ECA usage on all IADL areas included in this study. The results of the ANOVA tests are presented in Table 3. As the table shows, the extracted profiles differ significantly in all acceptability variables.

The post-hoc tests additionally revealed that the acceptability of ECAs in the area of communication differs significantly between profiles 1 and 2 (*p* = 0.002), 1 and 3 (*p* = 0.001), and 1 and 4 (*p* = 0.001). Acceptability of ECAs in the area of food differs significantly between profiles 1 and 4 (*p* = 0.035), while also only profiles 1 and 4 differ significantly in the acceptability of ECAs in the area of health (*p* = 0.045). The acceptability of ECAs in the area of purchasing differs significantly between profiles 2 and 4 (*p* = 0.019), and 3 and 4 (*p* < 0.001), while it approaches significance between profiles 1 and 4 (*p* = 0.077). The acceptability of ECAs in the area of finance differs significantly between profiles 1 and 4 (*p* < 0.001), and profiles 3 and 4 (*p* < 0.001). The acceptability of ECAs in the area of infotainment differs significantly between profiles 1 and 4 (*p* = 0.002), 2 and 3 (*p* < 0.001), and 2 and 4 (*p* < 0.001), while it approaches significance between profiles 1 and 3 (*p* = 0.058). Detailed results of post-hoc tests can be found in the Supplementary Materials.

## 4. Discussion

The present study aimed to investigate how aging adults perceive different IADLs. Additionally, our goal was to identify the latent profiles of ageing adults based on their perceived importance of various IADLs and compare them regarding their acceptance of ECAs in different life domains.

First, our study, conducted on a sample of Slovene older adults living in residential care settings, revealed which areas of daily living are perceived as highly important. The areas that are prioritized by aging adults are health, communication, and infotainment. These results are not particularly surprising, as aging adults are especially prone to disease and disability (e.g., [46]), as well as social isolation and loneliness (e.g., [47]). Since these areas represent the key challenges of aging, taking medicine as prescribed, going to the doctor, having conversations with relatives and friends via a phone or the internet, watching television shows and movies, and similar activities are very valuable because they keep these challenges under control or help reduce them. On the other hand, technology, managing money, shopping, and food seem to be less important in our sample, even though there are considerable differences between aging adults in their perceptions of these domains (reflected by relatively large standard deviations). This finding supports the notion that aging adults, even those who live in residential care settings where some tasks can be performed by the staff, have very diverse needs, preferences, and interests [4]. Hence, it is vital to identify the main groups of aging adults with similar needs and provide them with personalized technology that is relevant to them [9].

Second, the analyses revealed that aging adults can be assigned into one of the four profiles: a low motivation profile (low perceived importance of all IADLs), a high motivation profile (high perceived importance of all IADLs), and two selectively motivated profiles (one that primarily values health and one that primarily values technology, communication, and infotainment). Prevalence wise, more than half of participants (especially younger ones) find various areas of daily living important, about one-third of participants perceive some, but not all, areas as important, while only about a tenth of the sample finds IADLs uniformly unimportant. These results—combined with the fact that aging adults generally face impairments in various areas of functioning [5,10,11]—imply that aging adults may be motivated to achieve or sustain the ability to actively participate in various activities, although the specific activities differ between the profiles.

Third, aging adults’ willingness to accept ECAs differed between the identified latent profiles. Specifically, individuals who perceive different IADLs as more important are more likely to accept the ECAs in different life domains. Their readiness to accept ECAs coincides with their ratings of daily living areas; a high motivation profile is generally willing to use ECAs in all domains, selective motivation profiles only in certain domains, and a low motivation profile in none of the domains. This finding bears important implications for developing technology tailored to aging adults’ needs and may help bridge the gap between individuals’ expectations and needs and the currently available support services. In particular, acquiring data on the perceived importance of different IADLs is a relatively easy but important step that can inform the deployment of appropriate ECAs, as profile membership unravels the areas that groups of individuals find most important and the areas where they are likely to accept the help of ECAs.

### Limitations and Future Research

Our findings should be considered in light of some limitations. First of all, while the number of participants in research articles using latent profile analyses has a substantial range (e.g., [45]), and older people are generally challenging to recruit for research purposes (e.g., [48]), the present study has a relatively small sample size. As such, we encourage future research to replicate our results on a larger sample of aging adults living in residential facilities. Additionally, similar research questions could be extended to aging adults who still live at home or have other living arrangements.

Next, one of the main disadvantages of the study is its cross-sectional design, which makes it impossible to make any speculations regarding causality. The current study also lacks the practical evaluation of the identified profiles. To overcome this barrier, future studies could employ a longitudinal design and observe actual acceptance of technology over a more extended period. For example, researchers could investigate whether profile membership based on the importance of different IADLs established at time point 1 indeed predicts the actual adoption of digital technologies at time point 2 and prolonged use of such technologies at time point 3. Alternatively, future research could test the effects of matching the services provided by technology with the preferences of aging adults (based on profile membership) via controlled trials in natural environments.

Since this study was a part of larger data collection, most of the variables reported in this study had to be measured in a very concise and efficient manner, often using only a single item to capture individuals’ perception of different daily living areas and their acceptance of ECAs in different life domains. Future studies could hence pay particular attention to measuring these variables more extensively and with questionnaires that have previously been validated in this target group. Since our approach was quite broad and general, covering many areas of daily living, the next phase of research could be focused on specific areas aging adults find most important and go into more detail. For example, future research should explore not only the services that ECAs for aging adults should incorporate but also how these services should be incorporated.

## 5. Conclusions

Despite limitations, the findings of our study have important theoretical and practical implications for researchers and developers of digital assistive technology and ECAs specifically. In particular, our results clearly highlight that aging adults differ in areas they find most important and hence have different needs and preferences. Additionally, the results suggest that these needs and preferences interact and combine in specific ways, forming profiles of aging adults who are similar to each other and different from individuals who belong to other profiles. Taken together, these results provide a better understanding of what services ECAs need to provide to specific individuals based on their profile membership. The findings also support the broader initiative of taking a user-centered and personalized approach to developing new technology. We argue that this step is necessary to achieve higher adoption of ECAs in this target group and support aging adults in attaining a higher quality of life.

## Figures and Tables

**Table 1:** Means, standard deviations, and correlations between indicator variables.

	*M*	*SD*	1	2	3	4	5	6	7	8
1. Gender	0.66	0.48	-							
2. Age	81.42	7.84	0.38 **	-						
3. Technology imp.	7.05	2.41	−0.27 **	0.26 **	-					
4. Communication imp.	8.32	1.91	0.07	−0.03	0.45 **	-				
5. Food imp.	3.72	2.68	0.04	−0.15 *	0.21 **	0.18 *	-			
6. Health imp.	8.65	1.62	0.06	0.00	0.22 **	0.34 **	0.19 **	-		
7. Shopping imp.	4.23	2.71	−0.08	−0.12	0.15 *	0.19 *	0.35 **	0.14	-	
8. Managing money imp.	6.44	3.41	−0.13	−0.07	0.19 **	0.23 **	0.22 **	0.29 **	0.35 **	-
9. Infotainment imp.	8.04	1.89	−0.11	−0.12	0.40 **	0.24 **	0.15 *	0.09	0.18 *	0.14

Notes. Gender: 0 = male, 1 = female. * *p* < 0.050, ** *p* < 0.010.

**Table 2:** Fit indices for profile solutions.

Number of Profiles	*LL*	*FP*	*BIC*	*SABIC*	Entropy	*BLRT (p)*	Smallest Profile Size
2	−3561.74	28	7269.65	7180.97	0.971	<0.001 ***	11.35% (*n* = 21)
3	−3493.85	38	7186.07	7065.71	0.957	<0.001 ***	5.41% (*n* = 10)
4	−3465.77	48	7182.13	7030.09	0.931	<0.001 ***	9.19% (*n* = 17)
5	−3444.77	58	7192.33	7008.62	0.894	0.333	5.41% (*n* = 10)

Notes. *** *p* < 0.001.

**Table 3:** Differences between profiles regarding the acceptability of ECAs in different IADL domains.

	Profile 1 (*n* = 19)	Profile 2 (*n* = 17)	Profile 3 (*n* = 47)	Profile 4 (*n* = 102)	ANOVA	
	*M (SD)*	*M (SD)*	*M (SD)*	*M (SD)*	*df*	*F*
ECA idea—communication ^a^	2.92 (1.15)	4.15 (0.55)	4.21 (0.70)	4.26 (0.78)	(3, 178)	7.333 ***
ECA idea—food ^b^	2.05 (1.15)	2.29 (1.09)	2.41 (1.71)	2.92 (1.31)	(3, 181)	4.065 **
ECA idea—health ^b^	3.50 (0.87)	3.66 (0.98)	3.88 (0.82)	4.02 (0.67)	(3, 179)	3.121 *
ECA idea—purchasing ^b^	2.13 (1.28)	1.94 (0.88)	1.98 (1.18)	2.89 (1.25)	(3, 179)	8.097 ***
ECA idea—finance ^a^	1.42 (0.65)	1.94 (1.20)	1.52 (1.04)	2.64 (1.15)	(3, 181)	18.304 ***
ECA idea—infotainment ^b^	3.29 (1.12)	2.74 (1.21)	4.00 (1.08)	4.20 (0.90)	(3, 180)	13.208 ***

*Notes*. Degrees of freedom differ between the dependent variables due to missing data. ^a^ Welch’s ANOVA was used due to unequal variances exhibited by the data. ^b^ Classic ANOVA was used. * *p* < 0.050, ** *p* < 0.010, *** *p* < 0.001. Profile 1 = low motivation profile; Profile 2 = selective motivation profile with high importance of physical well-being; Profile 3 = selective motivation profile with high importance of psychological well-being; Profile 4 = high motivation profile.

## Data Availability

Aggregated data are available upon reasonable request.

## Supplementary Materials

The following are available online at https://www.mdpi.com/article/10.3390/ijerph19042373/s1, Table S1: Games-Howell’s post-hoc tests regarding the acceptability of ECAs in the communication domain, Table S2: Hochsberg’s post-hoc tests regarding the acceptability of ECAs in the food domain, Table S3: Hochsberg’s post-hoc tests regarding the acceptability of ECAs in the health domain, Table S4: Hochsberg’s post-hoc tests regarding the acceptability of ECAs in the shopping domain, Table S5: Games-Howell’s post-hoc tests regarding the acceptability of ECAs in the managing money domain, Table S6: Hochsberg’s post-hoc tests regarding the acceptability of ECAs in the infotainment domain.
