# Supplementary material for: Aging Adults’ Motivation to Use Embodied Conversational Agents in Instrumental Activities of Daily Living: Results of Latent Profile Analysis
Source: Int J Environ Res Public Health. 2022 Feb 18;19(4):2373. doi: 10.3390/ijerph19042373 (PMC8872482; doi:10.3390/ijerph19042373)
Supplement: Supplementary file 1 [file ijerph-19-02373-s001.zip › ijerph-1567433-supplementary.pdf]

Supplementary file

Table S1. Games-Howell's post-hoc tests regarding the acceptability of ECAs in the communication domain.

Profile A	Profile B	Mean difference (A – B)	*SE*	*p*	95 % CI
Profile 1	Profile 2	- 1.23	0.30	.002	[- 2.07, - 0.40]
	Profile 3	- 1.30	0.29	.001	[- 2.10, - 0.49]
	Profile 4	- 1.34	0.28	.001	[- 2.13, - 0.55]
Profile 2	Profile 3	- 0.07	0.17	.979	[- 0.52, 0.39]
	Profile 4	- 0.11	0.15	.897	[- 0.53, 0.32]
Profile 3	Profile 4	- 0.04	0.13	.988	[- 0.38, 0.29]

Notes. Profile 1 = low motivation profile; Profile 2 = selective motivation profile with high importance of physical well-being; Profile 3 = selective motivation profile with high importance of psychological well-being; Profile 4 = high motivation profile.

Table S2. Hochsberg's post-hoc tests regarding the acceptability of ECAs in the food domain.

Profile A	Profile B	Mean difference (A – B)	*SE*	*p*	95 % CI
Profile 1	Profile 2	- 0.24	0.41	.993	[- 1.34, 0.86]
	Profile 3	- 0.36	0.34	.863	[- 1.26, 0.54]
	Profile 4	- 0.68	0.31	.035	[- 1.69, - 0.40]
Profile 2	Profile 3	- 0.12	0.35	1.000	[- 1.05, 0.81]
	Profile 4	- 0.62	0.33	.295	[- 1.49, 0.24]
Profile 3	Profile 4	- 0.50	0.23	.129	[- 1.08, 0.08]

Notes. Profile 1 = low motivation profile; Profile 2 = selective motivation profile with high importance of physical well-being; Profile 3 = selective motivation profile with high importance of psychological well-being; Profile 4 = high motivation profile.

Table S3. Hochsberg's post-hoc tests regarding the acceptability of ECAs in the health domain.

Profile A	Profile B	Mean difference (A – B)	*SE*	*p*	95 % CI
Profile 1	Profile 2	- 0.16	0.26	.992	[- 0.85, 0.54]
	Profile 3	- 0.38	0.21	.355	[- 0.94, 0.18]
	Profile 4	- 0.52	0.19	.045	[- 1.04, - 0.01]
Profile 2	Profile 3	- 0.23	0.22	.885	[- 0.81, 0.36]
	Profile 4	- 0.37	0.20	.365	[- 0.91, 0.18]
Profile 3	Profile 4	- 0.14	0.13	.873	[- 0.50, 0.22]

Notes. Profile 1 = low motivation profile; Profile 2 = selective motivation profile with high importance of physical well-being; Profile 3 = selective motivation profile with high importance of psychological well-being; Profile 4 = high motivation profile.

Table S4. Hochsberg's post-hoc tests regarding the acceptability of ECAs in the shopping domain.

Profile A	Profile B	Mean difference (A – B)	*SE*	*p*	95 % CI
Profile 1	Profile 2	0.19	0.40	.998	[- 0.88, 1.26]
	Profile 3	0.15	0.33	.998	[- 0.72, 1.03]
	Profile 4	- 0.75	0.30	.077	[- 1.56, 0.05]
Profile 2	Profile 3	- 0.04	0.34	1.000	[- 0.95, 0.86]
	Profile 4	- 0.95	0.32	.019	[- 1.79, - 0.10]
Profile 3	Profile 4	- 0.91	0.21	.000	[- 1.48, - 0.34]

Notes. Profile 1 = low motivation profile; Profile 2 = selective motivation profile with high importance of physical well-being; Profile 3 = selective motivation profile with high importance of psychological well-being; Profile 4 = high motivation profile.

Table S5. Games-Howell's post-hoc tests regarding the acceptability of ECAs in the managing money domain.

Profile A	Profile B	Mean difference (A – B)	*SE*	*p*	95 % CI
Profile 1	Profile 2	- 0.52	0.33	.401	[- 1.42, 0.38]
	Profile 3	- 0.10	0.21	.965	[- 0.67, 0.47]
	Profile 4	- 1.22	0.19	.000	[- 1.72, - 0.71]
Profile 2	Profile 3	0.42	0.33	.583	[- 0.48, 1.32]
	Profile 4	- 0.70	0.31	.147	[- 1.56, 0.17]
Profile 3	Profile 4	-1.12	0.19	.000	[- 1.61, - 0.62]

Notes. Profile 1 = low motivation profile; Profile 2 = selective motivation profile with high importance of physical well-being; Profile 3 = selective motivation profile with high importance of psychological well-being; Profile 4 = high motivation profile.

Table S6. Hochsberg's post-hoc tests regarding the acceptability of ECAs in the infotainment domain.

Profile A	Profile B	Mean difference (A – B)	*SE*	*p*	95 % CI
Profile 1	Profile 2	0.55	0.33	.463	[- 0.34, 1.44]
	Profile 3	- 0.71	0.27	.058	[- 1.44, 0.01]
	Profile 4	- 0.91	0.25	.002	[- 1.58, - 0.25]
Profile 2	Profile 3	- 1.26	0.28	.000	[- 2.02, - 0.51]
	Profile 4	- 1.47	0.26	.000	[- 2.17, - 0.77]
Profile 3	Profile 4	- 0.20	0.18	.823	[- 0.67, 0.27]

Notes. Profile 1 = low motivation profile; Profile 2 = selective motivation profile with high importance of physical well-being; Profile 3 = selective motivation profile with high importance of psychological well-being; Profile 4 = high motivation profile.
